# Prevalence and Clinical Impact of Immune-Mediated Inflammatory Diseases in Patients with Inflammatory Bowel Disease: Results from a Large Retrospective Observational Study

**DOI:** 10.3390/jcm13041019

**Published:** 2024-02-09

**Authors:** Marta Vernero, Simone Saibeni, Davide Scalvini, Carolina Cicalini, Lorenzo Chiarello, Silvia Nardi, Davide Giuseppe Ribaldone, Cristina Bezzio

**Affiliations:** 1 Gastroenterology Department, Città della Salute e della Scienza Hospital, 10126 Torino, Italy; mvernero@cittadellasalute.to.it (M.V.); davidegiuseppe.ribaldone@unito.it (D.G.R.); 2 Gastroenterology Unit, ASST Rhodense, Rho Hospital, 20017 Rho, Italy; 3 Department of Medical Sciences, University of Pavia, 27100 Pavia, Italy; davide.scalvini01@universitadipavia.it (D.S.); carolina.cicalini01@universitadipavia.it (C.C.); 4 Department of Medical Sciences, University of Turin, 10124 Torino, Italy; lorenzo.chiarello@edu.unito.it (L.C.); silvia.nardi@edu.unito.it (S.N.); 5 IBD Centre, Humanitas Clinical and Research Centre, 20089 Rozzano, Italy; cristina.bezzio@hunimed.eu; 6 Department of Biomedical Sciences, Humanitas University, 20072 Milan, Italy

**Keywords:** IMID, IBD, Crohn’s disease, ulcerative colitis, clinical course, extraintestinal manifestations, quality of life

## Abstract

**(1) Background:** Inflammatory bowel diseases (IBDs) are chronic inflammatory disorders involving innate and adaptive immune responses. Despite primarily affecting the gut, recent insights highlight systemic implications, expanding our understanding beyond intestinal boundaries. **(2) Methods:** This retrospective multicentric study explored the association of IBD and immune-mediated inflammatory diseases (IMIDs) and the impact of concurrent IMIDs on the course of IBD. Clinical data were collected from consecutive medical records of patients with IBD. For assessing the impact of concurrent IMIDs, a control group of IBD patients without associated IMIDs was considered. **(3) Results:** Of 6589 IBD patients, 6.8% exhibited concomitant IMIDs. Notably, 79.8% of these patients had an aggressive disease course. Psoriasis, atopic dermatitis, and type 1 diabetes mellitus prevalence were lower in the IBD population than in the general population. Conversely, multiple sclerosis, primary sclerosing cholangitis, and pyoderma gangrenosum were more prevalent in IBD patients. Among the patients with a concomitant IMID, 79.8% had an aggressive disease course vs. 8.1% in the control group (*p* < 0.001). **(4) Conclusions:** This study underscores the frequency of IMIDs in IBD patients and their association with a more aggressive disease course. The recognition of concurrent IMIDs is crucial for comprehensive patient management, influencing therapeutic decisions and potentially improving outcomes.

## 1. Introduction

The inflammatory bowel diseases (IBDs), including ulcerative colitis (UC) and Crohn’s disease (CD), are chronic inflammatory diseases in which alterations of the innate and adaptive immune responses play a crucial role in starting and perpetuating intestinal inflammation [1]. In Italy and generally in the western world, they have a prevalence of between 180 and 300 cases/105 and are of growing incidence in developing countries [2]. Not a long time ago, a new concept was delineated: despite the fact that the inflammatory core remains in the gut, IBDs involve a crucial intertwining of circuits and relevant organ damage that can take place beyond the bowel boundaries [3]. The envisaging of IBD as a systemic disease has now been fostered by a group examining the extraintestinal manifestations (EIMs) of IBDs [4].

Indeed, in addition to gastrointestinal symptoms, these diseases may present some signs that refer to other body districts, and, in severe cases, systemically. The involvement of organs other than those of the gastrointestinal tract is called “extraintestinal manifestations”, which occur in different percentages of IBD patients; sometimes, a single patient has more than one extraintestinal manifestation [5]. Some extraintestinal manifestations of IBDs are related to intestinal inflammation, while others are independent of the disease course.

Recently, the European Crohn’s and Colitis Organisation (ECCO) defined the extraintestinal manifestations of IBDs as any “inflammatory pathology in a patient with IBD that is located outside the gut and for which the pathogenesis is either dependent on extension/translocation of immune responses from the intestine or is an independent inflammatory event perpetuated by IBD or that shares a common environmental or genetic predisposition with IBD” [6]. The most frequent locations of extraintestinal manifestations are the joints (peripheral and axial spondyloarthropathies), skin (erythema nodosum, pyoderma gangrenosum), eyes (uveitis, episcleritis, iridocyclitis), and hepatobiliary tract (primary sclerosing cholangitis) [5].

Paradoxical reactions to IBD treatment may also be considered part of the extraintestinal manifestations of IBDs. Moreover, some pathologies are more common in IBD patients, but no pathological link with IBDs has been established yet. Indeed, Hedin et al. [6] talked about “associated conditions with an uncertain mechanism”, among which almost every one was some kind of immune-mediated inflammatory disease (IMID). IMIDs are a heterogeneous group of apparently unrelated conditions involving common inflammatory pathways and pathogenic mechanisms. IMIDs include over 100 different conditions, one of which is IBD [7]. The exact cause of IMIDs is still unclear, but it has been postulated that IMIDs arise from the complex interactions between a susceptible genome and environmental factors (including antibiotics, smoking, infections), leading to alterations of the innate and adaptive immune systems that ultimately perpetuate chronic inflammation through an enhancement in the proinflammatory cascade [8].

About one-quarter of IBD patients have a concomitant IMID, while, in the general population, the total prevalence of these diseases is only about 5–7% [9,10,11]. In IBD patients, IMIDs mostly affect women and people with CD [11]. It has been estimated that the incidence rate in IBD patients is almost twice that in IBD-free patients [12]. IBD patients with concomitant IMIDs seem to have a more aggressive disease phenotype [9,12,13], with higher rates of surgery and treatment with anti-TNF agents, and, in UC, a more frequent pancolonic extent of the disease [12,13]. As observed in a Danish cohort [13], when the diagnosis of IMID preceded that of IBD, which happened in nearly 80% of cases, the clinical evolution was worse.

Particularly among the IBD population, dermatological manifestations are frequently reported tas associated extraintestinal diseases [2]. Among the dermatological manifestations, IBD is significantly associated with psoriasis; in particular, the association is statistically stronger for CD versus UC and is bidirectional. The prevalence of psoriasis in patients with CD and UC is 3.6% and 2.8%, respectively, while the prevalence of paradoxical psoriasis in IBD is 6.7%, stressing the impact of therapy with anti-TNF-α drugs on the development of this disease [3]. Another significant association is with atopic dermatitis (AD), which has a prevalence of about 20% in children and up to 3% of adults, while it is reported to be 27% in CD patients. The link between IBD and AD is bidirectional, both for CD as well as for UC. The risk of developing IBD is lower in AD patients undergoing systemic steroid therapy, while it is higher in those exposed to topical steroid therapy [4,5]. Hidradenitis suppurativa (HS) is often associated with IBD as well; its prevalence in the general population is still unclear. However, among patients with CD, the prevalence of HS is between 15 and 26%, while among HS patients, about 2.0% have Crohn’s disease [6,14,15].

Regarding the gastrointestinal IMIDs associated with IBDs, autoimmune hepatitis and primary sclerosing cholangitis have an unclear prevalence due to their frequent overlap [16], while granulomatous hepatitis is a separate disease with an unknown prevalence in the general population and an estimated prevalence in the IBD population of 1% [17].

Moreover, IMIDs create large healthcare and economic burdens given their increasing incidence but especially due to their chronic and progressively worsening course. Indeed, IMID patients often need potentially harmful therapies. Patients with IMIDs are also at high risk of psychological comorbidities such as depression and anxiety due to the chronicity of the condition; in addition, comorbidities may extend beyond the primary target organs and include infections, renal, and cardiovascular diseases, and neoplasms [18,19,20].

The association of IBDs with other IMIDs is so strong that some authors suggested that IBDs should be considered the gastrointestinal manifestation of IMIDs rather than seeing IMIDs as diseases associated with IBDs [21].

Keeping these concepts in mind may help the internist to suspect IBD disguised as an unusual EIM and the IBD caretaker to decide to call a second-line specialist to best help the patient. Indeed, EIMs are often more threatening or disabling than the underlying IBD itself.

The primary objective of this present study was to evaluate the prevalence of concomitant IMIDs in the IBD population. The secondary objectives were to compare the prevalence of IMIDs in the IBD population to that of the same IMIDs in the general population (as known in the literature), to evaluate whether concomitant IMIDs make the progression of the intestinal pathology more aggressive compared to that in a control group and whether the number of IMIDs influences the progression of IBD.

## 2. Materials and Methods

This was a retrospective observational multicentric study conducted in the IBD outpatient clinic of Città della Salute e della Scienza Hospital and the IBD ambulatory of ASST Rhodense Hospital, located in Turin and Rho, Italy, respectively. The inclusion criteria were a certain diagnosis of chronic inflammatory bowel disease according to the ECCO criteria [14], time elapsed between IBD diagnosis and the last follow-up of at least 6 months, and a certain diagnosis (documented in the medical record with tests or visits to the relevant specialist) of at least one concomitant IMID in addition to IBD (only for cases). The exclusion criterion was the loss of follow-up before 6 months after IBD diagnosis. All clinical charts of patients being followed-up in the two ambulatories were screened, and all the patients meeting inclusion and exclusion criteria were included in the study.

The investigated IMIDs were erythema nodosum, primary sclerosing cholangitis (alone or in overlap syndrome with autoimmune hepatitis), autoimmune hepatitis alone, bullous epidermolysis, ankylosing spondylitis, type 1 diabetes mellitus, systemic lupus erythematosus, pyoderma gangrenosum, hidradenitis suppurativa, Sjogren syndrome, vasculitis, alopecia, scleroderma, vitiligo, uveitis, interstitial pneumonia, autoimmune pancreatitis, rheumatoid arthritis, coeliac disease, psoriasis, multiple sclerosis, atopic dermatitis, spondyloarthritis, asthma, and granulomatous hepatitis. Among the IMIDs, we did not take into account autoimmune thyroid diseases, as they are not rare in the general population and are thus difficult to potentially associate with IBD, as their treatment usually does not include immunosuppressive or biological therapy.

Each IMID prevalence in our IBD population was compared to what is reported for the general population (when available in the literature). Conclusive data about the prevalences of hidradenitis suppurativa, interstitial pneumonia, scleroderma, and spondylitis were not available in the literature, so we were not able to perform this comparison.

The control group was selected from among a large database including all patients with IBD already included in other studies, after deleting all patients with some concomitant IMIDs (as they were included in case group). From among this database, we selected patients for whom the needed characteristics listed above had been recorded. For each included patient (for cases), the following data were collected: age, sex, diagnosis (Crohn’s disease, ulcerative colitis, or undefined IBD), smoking habit, disease location, perianal disease (yes or no), presence of any IMID in addition to IBD, number of steroid cycles needed in patients’ history (and how many of them for IBDs), number of advanced therapies (immunosuppressants, biological therapies, or small molecules) needed and how many for IBDs, need for surgery, number of surgeries, and reason for surgery.

Aggressive disease was defined as the need for more than two steroid cycles for IBDs, the need for immunosuppressors or target therapy for IBDs, the need for hospitalization for IBD relapse, the need for surgery, or the persistence of invalidating symptoms (diarrhea, abdominal pain, blood in stools, fever, nausea, or vomiting) for 12 months despite therapies. The presence of only one of these criteria was suggestive of aggressive disease [15]. To assess the impact of concurrent IMIDs, a control group of IBD patients without associated IMIDs was considered. For the control group, data about age, sex, diagnosis (Crohn’s disease, ulcerative colitis, or undefined IBD), smoking habits, disease location, and perianal disease were collected.

The study protocol was approved by the Città della Salute e della Scienza Hospital ethical committee. The data were all collected in a database built in Excel 365 and analyzed using the MedCalc software version 22.005-© 2023, MedCalc Software Ltd (Ostend, Belgium). For quantitative variables, percentage frequencies were calculated and, when necessary, compared using the chi-squared test. For qualitative variables, normality was tested using the D’Agostino–Pearson test. In case of normality, the mean with the relative 95% confidence interval was calculated, while in case of non-normal distribution, the median with the relative interquartile range (25–75%) was calculated. For normally distributed variables, comparisons between means were made using Student’s *t*-test for independent samples, whereas for non-normally distributed variables, the medians were compared using the Mann–Whitney test for independent samples. The influence of variables was tested by means of logistic regression. Variables with a statistically significant influence on the outcome of the univariate analysis were included in the multivariate analysis. Statistical significance was set as *p*-values less than 0.005.

## 3. Results

A total of 6589 charts were evaluated. Among them, 451 (6.8%) patients with IBD and at least one other IMID were detected. Table 1 summarizes the basal characteristics of the population under study (IBD and IMID population). Most of the patients were diagnosed with CD (57.42%), and only 33.48% were men. Of note, perianal disease was present in nearly 25% of patients for who these data were available.

Among our population of patients with IBD and at least one other IMID, 298 (66.1%) had just one other IMID, 96 (21.3%) patients had two other IMID, 26 (5.7%) patients had three, and 31 (6.9%) patients have four IMIDs in addition to IBD. Table 2 shows the percentage of each IMID among the case (IBD and IMID) population. Of note, the most frequent IMID were erythema nodosum (22.8% of IMID associated with IBD), psoriasis (11.8%), peripheral arthritis (10.64%), rheumatoid arthritis (9.3%), and celiac disease (7.76%). Of note, we did not detect any cases of granulomatous hepatitis. Table 2 shows the prevalence of each detected IMID among our IBD population with at least one other IMID.

### 3.1. Comparison of Prevalence of IMIDs in Our Population vs. General Population

Table 3 shows the prevalence of each IMID in our IBD sample compared to what is reported for the general population. In our population, psoriasis had a lower prevalence than in the general population (0.7% vs. 2.8% *p* < 0.001), as did atopic dermatitis (0.1% vs. 1%; *p* < 0.001), type 1 diabetes mellitus (0.007% vs. 0.5%; *p* = 0.002), and vitiligo (0.03% vs. 0.7%; *p* < 0.001). On the other hand, multiple sclerosis prevalence was higher than in the general population (0.7% vs. 0.1% *p* < 0.001), as was that of erythema nodosum (1.53% vs. 0.003%; *p* < 0.001), primary sclerosing cholangitis (1% vs. 0.01%; *p* < 0.001), systemic lupus erythematosus (0.2% vs. 0.03%; *p* < 0.001), pyoderma gangrenosum (0.42% vs. 0.0005%; *p* > 0.001), and Sjogren syndrome (0.12% vs. 0.01%; *p* = 0.02).

### 3.2. Influence of the Presence of Concomitant IMIDs on Disease Course

For this outcome, a control group was selected among a database including all IBD patients included in a recent previous observational study after removing patients already included in the case group and patients for whom the needed characteristics had not been recorded. In the end, we selected 74 control patients. The basal characteristics of this population are summarized in Table 4.

**Figure 1:**
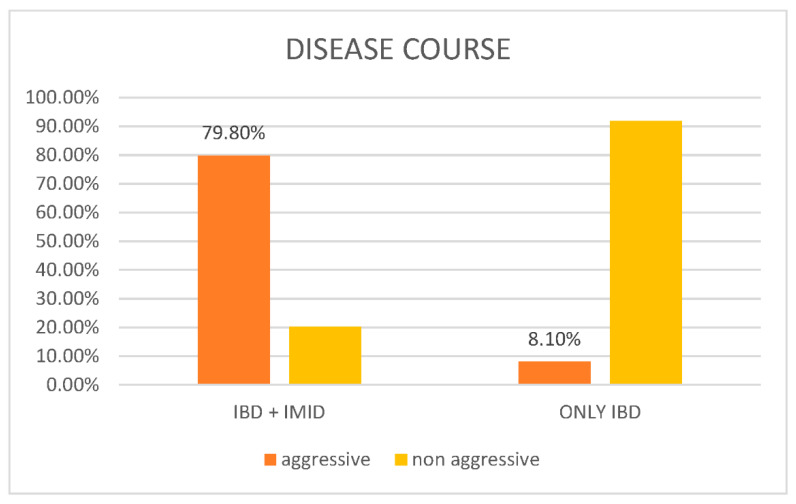
Graphical representation of disease course in patients with IBD and at least one concomitant IMID (cases) vs. patients with only IBD (control).

As reported in Figure 1, according to the Siegel criteria, 360 out of 451 cases (79.8%) had aggressive disease, and 91 patients out of 451 (20.2%) had nonaggressive disease. Among the controls, 6/74 (8.1%) had aggressive disease, while 68/74 (91.9%) had nonaggressive disease. The *p* value was lower than 0.001.

### 3.3. Influence of the Number of IMIDs on the Outcome

Logistic regression showed a significant influence of the number of IMIDs on disease activity, with an odds ratio (OR) of 1.6 (95% CI 1.02–2.67) and a significant *p* value (*p* = 0.02).

## 4. Discussion

This was an observational retrospective study on the prevalence of IMIDs in the IBD population. In our extensive cohort of patients with IBDs, 6.8% had a concomitant IMID, which is in line with what is reported in the literature [30]. However, even though IMIDs tend to be associated with other IMIDs, and despite the fact that IBD is an IMID itself, not all IMIDs have a significantly higher prevalence in the IBD population than in the general population. For instance, psoriasis has a prevalence of 1% in the general population, while in our IBD population, the prevalence of psoriasis was about 0.1%. Similarly, type 1 diabetes mellitus and vitiligo also had lower prevalences in our IBD population than in the general population. Conversely, multiple sclerosis is more prevalent in the IBD population than in the general population, as re primary sclerosing cholangitis, pyoderma gangrenosum, Sjogren syndrome, and systemic lupus erythematosus. In summary, some other IMIDs do not have a significantly higher or lower prevalence in patients with IBD than in the general population, particularly, rheumatoid arthritis, celiac disease, ankylosing spondylitis, and alopecia areata.

The actual prevalence of the IMIDs associated with IBDs is not known because a limited number of studies have investigated this association, and the results of these studies are partially conflicting. The low prevalence of certain IMIDs may be associated with the lack of direct investigation of these pathologies by the physician during the taking of the medical history. In this regard, we emphasize that we did not detect any case of asthma or granulomatous hepatitis. Indeed, in the past, the association of these diseases with IBDs and the potential impact of this association on the patients’ quality of life and disease course were not well known. It is also possible that patients did not report the presence of these concurrent diseases unless specifically queried by the physician, especially for the milder forms with a limited impact on quality of life. Differently, for other conditions such as type 1 diabetes and multiple sclerosis, the data could be more accurate, given their clinical and therapeutic relevance, and, therefore, there is increased attention from both physicians and patients.

One of the major findings of this study is that the presence of other IMIDs in addition to IBD influences the outcome of the IBD itself. Indeed, in 80% of patients, the IBD presented in an aggressive form, while in the control group, only 8.1% of patients had aggressive disease. Moreover, the multivariate analysis showed that the presence of at least one other IMID improves the ratio of aggressivity of the disease (OR 1.6). It is important to point out that aggressive disease was evaluated with reference to IBD; in other words, we considered the following items for the definition of aggressive IBD: severe symptoms for more than 12 months, surgery, the need for hospitalization, the need for more than two systemic steroid therapies, the need for immunosuppressors, or the need for biological therapy. For the last two items, only steroids and immunosuppressors or biological therapies prescribed for IBD were considered. The co-occurrence of IBD and IMID is associated with a worse course of intestinal disease, consistent with the existing literature data [9,12,13]. This result emphasizes the need for gastroenterologists to manage IBD by taking a more thorough medical history regarding concurrent IMIDs. In fact, in addition to being associated with a worse disease course, the presence of IMIDs is also linked to a stronger impact on the patients’ quality of life [9]. Moreover, recently, new drugs for the treatment of IBDs have been approved, and some of these are already being used for the management of other IMIDs. In this regard, the presence of a concurrent IMID could guide the gastroenterologist in choosing an appropriate medication for the treatment of both conditions.

This was a study on a large IBD population taking into account a large number of IMIDs; however, it had some limitations. First of all, its retrospective nature has the intrinsic problem of possible missing data, as they may not have been reported. Recall bias may have been further increased by the fact that some IMIDs can have a mild course when associated with IBD (e.g., psoriasis in our study) or by the fact that the presence of IMIDs was not systematically ascertained during the follow-up, potentially due a lower sensitivity to this issue in the past years and/or to the different sensitivity to this issue by the several physicians treating that patient. The limitations of our study are common to those that have retrospectively assessed the prevalence of immune-mediated inflammatory diseases (IMIDs) in patients with inflammatory bowel disease (IBD). Moreover, for some IMIDs, the higher prevalence is uncertain, and, for others, the data are not consistent and sometimes outright conflicting. Moreover, although our study was multicentric, it involved only two Italian IBD centers with a high number of patients and working as a third-level hospital for IBD treatment. So, the data may not be generalizable to other centers managing IBD patients or to other countries. Therefore, a prospective study is necessary to systematically evaluate the presence of specific IMIDs, whether they are common or rare and whether they are more or less associated with IBDs.

Our study highlights the importance of the multidisciplinary management of IBD patients, as these patients are more prone to developing other immune-mediated diseases that can influence the disease course. Indeed, we believe that IBD should not be considered a gut-limited disease but a manifestation of systemic inflammation involving the gut, possibly extending to other organs.

## 5. Conclusions

The immunological response at systemic and intestinal levels is the result of the composite interactions between environment, intestinal milieu, and genetics. The complex relations among these components may determine alterations in different pathways, eventually leading to pathological conditions such as IMIDs.

In recent years, we have observed a relevant improvement in the knowledge about the normal functions of and the pathological changes in immune systems, which led to the development of several new therapies with different mechanisms of action. Moreover, the roles of concomitant IMIDs, the reciprocal clinical influence, as well as the deleterious impact on quality of life and other chronic conditions have been better defined.

Our study assessed the frequency of IMIDs in IBD patients and their association with a more aggressive disease course. When a subject shows several concomitant IMIDs, there is a need for sequential multi- and interdisciplinary management in order to define a common and shared strategy considering all disease manifestations, instead of each IMID individually. The final aims of this approach are to avoid diagnostic delay, which has positive relevant implications through a prompt and correct diagnosis of IMID, and to integrate the assessment and treatment of IMIDs, warranting the right therapy (type and duration), the prevention of complications, and the improvement in clinical outcomes and quality of life.

In this direction, large prospective studies are essential to better define the real prevalence of IMIDs in the IBD population and their clinical correlates.

## Figures and Tables

**Table 1:** Basal characteristics of the population.

Diagnosis	CD *n* (%)	259 (57.4%)
	UC *n* (%)	151 (33.4%)
	IBD-U *n* (%)	41 (9.1%)
Sex (M)	*n* (%)	175 (38.8%)
Smoking	Never *n* (%)	139 (30.8%)
	Former *n* (%)	67 (14.9%)
	Active *n* (%)	51 (11.3%)
	Unknown *n* (%)	194 (43.0%)
UC extension CD localization	E1 *n* (%)	13 (9.6%)
	E2 *n* (%)	59 (43.4%)
	E3 *n* (%)	64 (47.1%)
	L1 *n* (%)	96 (37.1%)
	L2 *n* (%)	90 (34.7%)
	L3 *n* (%)	91 (35.1%)
	L4 *n* (%)	24 (9.3%)
	Unknown	15
Perianal disease	Available in 248 patients *n* (%)	105 (24.6%)
Age	Mean (SD)	55.45 (±15.4)

**Table 2:** Proportion of the detected IMIDs in our population of cases (IBD + one other IMID).

IMID	Proportion of IMID	%
Rheumatoid Arthritis	42/451	9.3%
Celiac Disease	35/451	7.8%
Psoriasis	50/451	11.1%
Multiple Sclerosis	10/451	2.2%
Atopic Dermatitis	12/451	2.7%
Axial Arthritis	6/451	1.3%
Peripheral Arthritis	48/451	10.6%
Erythema Nodosum	103/451	22.8%
Type 1 Diabetes Mellitus	5/451	1.1%
Systemic Lupus Erythematosus	13/451	2.9%
Pyoderma Gangrenosum	28/451	6.2%
Hidradenitis Suppurativa	15/451	3.3%
Sjogren Syndrome	8/451	1.8%
Vasculitis	9/451	2,0%
Alopecia Areata	2/451	0.4%
Scleroderma	2/451	0.4%
Vitiligo	2/451	0.4%
Uveitis	4/451	0.9%
Interstitial Pneumonia	1/451	0.2%
Autoimmune Pancreatitis	2/451	0.4%

**Table 3:** Comparison of the prevalence of IMIDs in IBD population and in general population; “-” indicates that the prevalence of IMIDs in the general population was not available in the literature.

**IMID**	**Prevalence in IBDs** ***n* (%)**	**Prevalence in General Population** ***n* (%)**	**Significance Level** **(*p*)**
Rheumatoid Arthritis	42/6589 (0.6%)	1/250 (0.4%) [22]	0.7
Celiac Disease	35/6589 (0.5%)	1/300 (0.33%) [23]	0.67
Psoriasis	50/6589 (0.7%)	28/1000 (2.8%) [24]	<0.001
Multiple Sclerosis	10/6589 (0.7%)	113/100,000 (0.1%) [25]	<0.001
Atopic Dermatitis	12/6589 (0.1%)	10/1000 (1%) [26]	<0.001
Axial Arthritis	6/6589 (0.01%)	-	-
Peripheral Arthritis	48/6589 (0.24%)	-	-
Erythema Nodosum	103/6589 (1.53%)	3/100,000 (0.003%) [27]	<0.001
Primary Sclerosing Cholangitis	66/6589 (1%)	9/100,000 (0.01%) [28]	<0.001
Ankylosing Spondylitis	30/6589 (0.45%)	1/100 (1%) [29]	0.47
Type 1 Diabetes Mellitus	5/6589 (0.07%)	1/200 (0.5%) [30]	0.002
Systemic Lupus Erythematosus	13/6589 (0.2%)	27/71,204 (0.03%) [31]	<0.001
Pyoderma Gangrenosum	28/6589 (0.42%)	5.17/100,000 (0.0005%) [32]	<0.001
Hidradenitis Suppurativa	15/6589 (0.23%)	-	-
Sjogren Syndrome	8/6589 (0.12%)	1–5/100,000 (0.01%) [33]	0.02
Vasculitis	9/6589 (0.14%)	-	-
Alopecia Areata	2/6589 (0.03%)	1–5/10,000 (0.03%) [34]	0.98
Scleroderma	2/6589 (0.03%)	-	-
Vitiligo	2/6589 (0.03%)	7/1000 (0.7%) [35]	<0.001
Uveitis	4/6589 (0.06%)	-	-
Interstitial Pneumonia	1/6589 (0.015%)	-	-
Autoimmune Pancreatitis	2/6589 (0.03%)	-	-

**Table 4:** Basal characteristics of control population.

Diagnosis	CD *n* (%)	35 (47.3%)
	UC *n* (%)	36 (48.6%)
	IBD-U *n* (%)	3 (4.1%)
Sex (M)	*n* (%)	33 (44.6%)
Smoking	Never *n* (%)	42 (56.8%)
	Former *n* (%)	11 (14.9%)
	Active *n* (%)	21 (28.4%)
Age	Mean (SD)	53.8 (±13.6)

## Data Availability

Data were collected and preserved by the corresponding authors and are available upon request.

## References

[1] Harbord M., Annese V., Vavricka S.R., Allez M., Barreiro-de Acosta M., Boberg K.M., Burisch J., De Vos M., De Vries A.-M., Dick A.D. (2016). The First European Evidence-based Consensus on Extra-intestinal Manifestations in Inflammatory Bowel Disease. J. Crohn’s Colitis.

[2] Gordon H., Burisch J., Ellul P., Karmiris K., Katsanos K., Allocca M., Bamias G., Barreiro-de Acosta M., Braithwaite T., Greuter T. (2023). ECCO Guidelines on Extraintestinal Manifestations in Inflammatory Bowel Disease. *J. Crohn’s Colitis*. https://pubmed.ncbi.nlm.nih.gov/37351850/.

[3] Yates V.M., Watkinson G., Kelman A. (1982). Further evidence for an association between psoriasis, Crohn’s disease and ulcerative colitis. Br. J. Dermatol..

[4] Egeberg A., Mallbris L., Warren R.B., Bachelez H., Gislason G.H., Hansen P.R., Skov L. (2016). Association between psoriasis and inflammatory bowel disease: A Danish nationwide cohort study. Br. J. Dermatol..

[5] Nutten S. (2015). Atopic dermatitis: Global epidemiology and risk factors. Ann. Nutr. Metab..

[6] Jemec G.B.E. (2012). Clinical practice. Hidradenitis suppurativa. N. Engl. J. Med..

[7] Tavares Da Silva F., De Keyser F., Lambert P.H., Robinson W.H., Westhovens R., Sindic C. (2013). Optimal approaches to data collection and analysis of potential immune mediated disorders in clinical trials of new vaccines. Vaccine.

[8] Rahman P., Inman R.D., El-Gabalawy H., Krause D.O. (2010). Pathophysiology and pathogenesis of immune-mediated inflammatory diseases: Commonalities and differences. J. Rheumatol. Suppl..

[9] Conway G., Velonias G., Andrews E., Garber J.J., Yajnik V., Ananthakrishnan A.N. (2017). The impact of co-existing immune-mediated diseases on phenotype and outcomes in inflammatory bowel diseases. Aliment. Pharmacol. Ther..

[10] Kuek A., Hazleman B.L., Ostör A.J.K. (2007). Immune-mediated inflammatory diseases (IMIDs) and biologic therapy: A medical revolution. Postgrad. Med. J..

[11] GBD 2016 Disease and Injury Incidence and Prevalence Collaborators (2017). Global, regional, and national incidence, prevalence, and years lived with disability for 328 diseases and injuries for 195 countries, 1990–2016: A systematic analysis for the Global Burden of Disease Study 2016. Lancet Lond. Engl..

[12] Wilson J.C., Furlano R.I., Jick S.S., Meier C.R. (2016). Inflammatory Bowel Disease and the Risk of Autoimmune Diseases. J. Crohn’s Colitis.

[13] Burisch J., Jess T., Egeberg A. (2019). Incidence of Immune-Mediated Inflammatory Diseases Among Patients with Inflammatory Bowel Diseases in Denmark. Clin. Gastroenterol. Hepatol. Off. Clin. Pract. J. Am. Gastroenterol. Assoc..

[14] Ingram J.R., Jenkins-Jones S., Knipe D.W., Morgan C.L.I., Cannings-John R., Piguet V. (2018). Population-based Clinical Practice Research Datalink study using algorithm modelling to identify the true burden of hidradenitis suppurativa. Br. J. Dermatol..

[15] Tiri H., Jokelainen J., Timonen M., Tasanen K., Huilaja L. (2018). Somatic and psychiatric comorbidities of hidradenitis suppurativa in children and adolescents. J. Am. Acad. Dermatol..

[16] Bezzio C., Della Corte C., Vernero M., Di Luna I., Manes G., Saibeni S. (2022). Inflammatory bowel disease and immune-mediated inflammatory diseases: Looking at the less frequent associations. Ther. Adv. Gastroenterol..

[17] Hepatopancreatobiliary Manifestations and Complications Associated with Inflammatory Bowel Disease. https://pubmed.ncbi.nlm.nih.gov/20198712/.

[18] Baena-Díez J.M., Garcia-Gil M., Comas-Cufí M., Ramos R., Prieto-Alhambra D., Salvador-González B., Elosua R., Dégano I.R., Peñafiel J., Grau M. (2018). Association between chronic immune-mediated inflammatory diseases and cardiovascular risk. Heart Br. Card. Soc..

[19] Marrie R.A., Walld R., Bolton J.M., Sareen J., Walker J.R., Patten S.B., Singer A., Lix L.M., Hitchon C.A., El-Gabalawy R. (2017). Increased incidence of psychiatric disorders in immune-mediated inflammatory disease. J. Psychosom. Res..

[20] Beyaert R., Beaugerie L., Van Assche G., Brochez L., Renauld J.-C., Viguier M., Cocquyt V., Jerusalem G., Machiels J.-P., Prenen H. (2013). Cancer risk in immune-mediated inflammatory diseases (IMID). Mol. Cancer.

[21] Bezzio C., Alimenti E., Saibeni S. (2022). Letter: Immune-mediated inflammatory diseases and inflammatory bowel disease-are we ready for a Copernican revolution?. Aliment. Pharmacol. Ther..

[22] Fondazione Umberto Veronesi Artrite Reumatoide. https://www.fondazioneveronesi.it/magazine/tools-della-salute/glossario-delle-malattie/artrite-reumatoide.

[23] EpiCentro Celiachia Aspetti Epidemiologici in Italia. https://www.epicentro.iss.it/celiachia/epidemiologia-italia.

[24] Salute M della Linea Guida Sulla Psoriasi, Presentato Aggiornamento Coordinato dall’ISS. https://www.salute.gov.it/portale/news/p3_2_1_1_1.jsp?menu=notizie&p=dalministero&id=1072.

[25] EpiCentro Sclerosi Multipla Epidemiologia. https://www.epicentro.iss.it/sclerosi-multipla/epidemiologia.

[26] Bylund S., Kobyletzki L.B., Svalstedt M., Svensson Å. (2020). Prevalence and Incidence of Atopic Dermatitis: A Systematic Review. Acta Derm. Venereol..

[27] Jameson J.L., Fauci A., Kasper D., Hauser S., Longo D., Loscalzo J. (2017). Harrisons Principles of Internal Medicine.

[28] RISERVATIIUTID Orphanet: Colangite Sclerosante Primitiva. https://www.orpha.net/consor4.01/www/cgi-bin/Disease_Search.php?lng=IT&data_id=783&Disease_Disease_Search_diseaseGroup=colangite-sclerosante-primitiva&Disease_Disease_Search_diseaseType=Pat&Malattia(e)/%20gruppo%20di%20malattie=Colangite-sclerosante-primitiva&title=Colangite%20sclerosante%20primitiva&search=Disease_Search_Simple.

[29] Braun J., Sieper J. (2007). Ankylosing spondylitis. Lancet.

[30] Salute M della 14 Novembre 2022, Giornata Mondiale del Diabete. https://www.salute.gov.it/portale/nutrizione/dettaglioNotizieNutrizione.jsp?lingua=italiano&menu=notizie&p=dalministero&id=6064.

[31] Benucci M., Del Rosso A., Li Gobbi F., Manfredi M., Cerinic M.M., Salvarani C. (2005). Systemic lupus erythematosus (SLE) in Italy: An Italian prevalence study based on a two-step strategy in an area of Florence (Scandicci-Le Signe). Med. Sci. Monit. Int. Med. J. Exp. Clin. Res..

[32] Monari P., Moro R., Motolese A., Misciali C., Baraldi C., Fanti P.A., Caccavale S., Puviani M., Olezzi D., Zampieri P. (2018). Epidemiology of pyoderma gangrenosum: Results from an Italian prospective multicentre study. Int. Wound J..

[33] RISERVATIIUTID Orphanet: Sindrome di Sjögren Primitiva. https://www.orpha.net/consor/cgi-bin/OC_Exp.php?lng=IT&Expert=289390.

[34] MSD|Salute Alopecia Areata: Epidemiologia e Dati Demografici da uno Studio Inglese. https://msdsalute.it/approfondimenti/notizie/alopecia-areata-epidemiologia-e-dati-demografici-da-uno-studio-inglese/.

[35] Gandhi K., Ezzedine K., Anastassopoulos K.P., Patel R., Sikirica V., Daniel S.R., Napatalung L., Yamaguchi Y., Baik R., Pandya A.G. (2022). Prevalence of Vitiligo Among Adults in the United States. JAMA Dermatol..

